# *In Vivo* Antiplasmodial Activity of *Terminalia mantaly* Stem Bark Aqueous Extract in Mice Infected by *Plasmodium berghei*

**DOI:** 10.1155/2020/4580526

**Published:** 2020-06-29

**Authors:** Mariscal Brice Tchatat Tali, Cedric Derick Jiatsa Mbouna, Lauve Rachel Yamthe Tchokouaha, Patrick Valere Tsouh Fokou, Jaures Marius Tsakem Nangap, Rodrigue Keumoe, Alvine Ngoutane Mfopa, Issakou Bakarnga-via, Raceline Gounoue Kamkumo, Fabrice Fekam Boyom

**Affiliations:** ^1^Antimicrobial & Biocontrol Agents Unit, Laboratory for Phytobiochemistry and Medicinal Plants Study, Faculty of Science, University of Yaoundé 1, PO Box 812, Yaoundé, Cameroon; ^2^Institute for Medical Research and Medicinal Plants Studies (IMPM), Yaoundé, PO Box 6163, Yaoundé, Cameroon; ^3^Department of Biochemistry, Faculty of Science, University of Bamenda, PO Box 39, Bambili, Bamenda, Cameroon; ^4^Laboratory of Animal Physiology, Department of Animal Biology and Physiology, Faculty of Science, University of Yaoundé 1, PO Box 812, Yaoundé, Cameroon; ^5^Department of Biomedical and Pharmaceutical Sciences, Faculty of Human Health Sciences, University of Ndjamena, PO Box 1117, Ndjamena, Chad

## Abstract

**Background:**

*Terminalia mantaly* is used in Cameroon traditional medicine to treat malaria and related symptoms. However, its antiplasmodial efficacy is still to be established.

**Objectives:**

The present study is aimed at evaluating the *in vitro* and *in vivo* antiplasmodial activity and the oral acute toxicity of the *Terminalia mantaly* extracts.

**Materials and Methods:**

Extracts were prepared from leaves and stem bark of *T. mantaly*, by maceration in distilled water, methanol, ethanol, dichloromethane (DCM), and hexane. All extracts were initially screened *in vitro* against the chloroquine-resistant strain W2 of *P. falciparum* to confirm its *in vitro* activity, and the most potent one was assessed in malaria mouse model at three concentrations (100, 200, and 400 mg/kg/bw). Biochemical, hematological, and histological parameters were also determined.

**Results:**

Overall, 7 extracts showed *in vitro* antiplasmodial activity with IC_50_ ranging from 0.809 *μ*g/mL to 5.886 *μ*g/mL. The aqueous extract from the stem bark of *T. mantaly* (*Tm*sb^w^) was the most potent (IC_50_ = 0.809 *μ*g/mL) and was further assessed for acute toxicity and efficacy in *Plasmodium berghei*-infected mice. *Tm*sb^w^ was safe in mice with a median lethal dose (LD_50_) higher than 2000 mg/kg of body weight. It also exerted a good antimalarial efficacy *in vivo* with ED_50_ of 69.50 mg/kg and had no significant effect on biochemical, hematological, and histological parameters.

**Conclusion:**

The results suggest that the stem bark extract of *T. mantaly* possesses antimalarial activity.

## 1. Introduction

Malaria parasites caused more human deaths and diseases than all other eukaryotic pathogens combined [1]. In fact, there were about 228 million cases of malaria worldwide and 405 000 malaria deaths in 2018 with children under 5 years of age still the most vulnerable group affected [2]. In spite of being manageable, malaria continues to exert a heavy toll on mankind. The disease disproportionately affects the poor and disadvantaged people, who have limited access to health facilities and can barely afford the recommended treatment in most countries [2]. Cameroon continues to be a malaria endemic country where it is the leading cause of morbidity and mortality among the most vulnerable groups [3]. Artemisinin-based combination therapy has significantly reduced malaria morbidity and mortality. However, recently reported failures in treatment and parasite resistances [4], coupled with the fact that the cost of the current antimalarial drugs is prohibitive in poor settings, stress the urgent need for new safe and affordable antimalarial agents.

Plant products continue to make an immense contribution to malaria chemotherapy, either directly as antimalarial phytomedicine or as an important source of lead compounds for the discovery of new and potent antimalarial drugs [1, 5]. In Cameroon, plant extracts are still widely used to combat malaria and several other diseases in daily practices, especially in areas where access to standard treatments is limited [6, 7]. The rich and diverse Cameroonian flora is a potential reservoir of potent antiplasmodial natural compounds, and its exploration based on ethnopharmacological approach is still promising in the fight against malaria. Indeed, *Terminalia mantaly* (*Combretaceae*) is a plant of the Cameroonian pharmacopeia used for malaria and/or related symptoms [8–12]. *In vitro* and *in vivo* screening of such plant extracts for inhibitory activity against malaria parasites is the first step in the search for new natural plant-derived antimalarial leads. We previously reported the *in vitro* antiplasmodial activity of crude extracts from the leaves and stem bark of *T. mantaly* [13] against chloroquine-sensitive (*Pf3D7*) and chloroquine-resistant (*PfINDO*). However, no efficacy study in an animal model of malaria has been carried out so far. Therefore, the present study is aimed at confirming the safety and efficacy of its use.

## 2. Material and Methods

### 2.1. Plant Collection and Identification

*Terminalia mantaly* H. Perrier was harvested in August and September 2014 in Ngoa-Ekelle, Yaoundé, Cameroon. It was identified at the National Herbarium of Cameroon, Yaoundé, where voucher specimens are deposited under the registration number 64212/HNC.

### 2.2. Experimental Animals

Studies were conducted with healthy young, nulliparous, and nonpregnant Swiss albino mice female aged eight weeks weighing ≈20 g. These animals were bred in the animal house of the Laboratory of Pharmacology and Toxicology, Faculty of Medicine and Biomedical Sciences of University of Yaoundé 1. Animals were maintained at room temperature on a 12 h light-dark natural cycle and fed using conventional laboratory diets with an unlimited supply of drinking water. All animal procedures were conducted according to relevant national and international guidelines. The protocol received approval from the Institutional Review Board (IRB No. 001/UY11 BTC/IRBI 2009), Biotechnology Centre, University of Yaoundé 1, Cameroon.

### 2.3. Extract Preparation

Leaves and stem bark from *T. mantaly* were air-dried and ground into fine powder using an electric dry mill (Hammer Mill, Leabon 9FQ). A total amount of 100 g of the powder from each part was soaked in 1000 mL of distilled water, methanol, 70% (hydroethanol) and 95% ethanol, dichloromethane (DCM), and hexane for 72 hours at room temperature. The mixtures were filtered and evaporated using a rotary evaporator (Rotavapor, BUCHI 071). The aqueous extracts were lyophilized in the Laboratory of Phytochemistry, Institute for Medical Research and Medicinal Plants Studies (IMPM), Yaoundé, Cameroon, using a Lyophilizer (Virtis Wizard 2.0 Freeze Dryer, XLS-70).

### 2.4. Phytochemical Analysis

Crude extracts were further screened for their content in different classes of secondary metabolites including alkaloids, phenolics, glucosides, triterpenes, saponins, tannins, and flavonoids using standard techniques [14–16].

### 2.5. In Vitro Antiplasmodial Activity Confirmation

*Pf*W2, known to be resistant to chloroquine and other antimalarials [17], were cultured into sealed flasks at 37°C, in a 3% O_2_, 5% CO_2_, and 91% N_2_ atmosphere in RPMI 1640, 25 mM HEPES, pH 7.4, supplemented with heat-inactivated 10% human serum and human erythrocytes to achieve 2% hematocrit. Parasites were synchronized in the ring stage by serial treatment with 5% sorbitol (Sigma, Taufkirchen, Germany) [18] and tested at 1% parasitemia. Stock solutions of plant extracts were prepared at 1 mg/mL in DMSO, diluted from 10 *μ*g/mL as needed for individual experiments, and tested in triplicate as described previously by Yamthe et al. [7]. The most potent antiplasmodial extract, the aqueous extract from *T. mantaly* stem bark (*Tm*sb^w^), was then submitted to acute toxicity study and *in vivo* antimalarial activity.

### 2.6. Acute Toxicity of Aqueous Extract from *T. mantaly* Stem Bark (*Tm*sb^w^)

The acute toxicity of *Tm*sb^w^ was evaluated according to the Organization for Economic Cooperation and Development (OECD 423) protocol briefly described below, with some modifications [19]. Animals in wire mesh bottom cages were starved for 12 h prior to the experiment and had free access to water. Six female mice were used for experimentation, divided into two groups of three animals.

For the study, a dose of extract at 2000 mg/kg of body weight was administrated to the three animals of the test group. The three other animals of the control group received distilled water at dose 10 mL/kg. After administration, animals were observed for general behavior changes and mortality continuously for 30 min and thereafter intermittently for 4 h and 24 h later. During observation times, behavioral parameters such as piloerection, appearance of faces, sensitivity to sound and touch, mobility, aggression, and mortality were recorded. Mice were further observed for up to 14 days postadministration during which mortality, body weights, and gross behavioral changes were recorded daily. At day 15 posttreatment, animals were sacrificed and the macroscopic appearance of organs such as the liver, kidneys, heart, and lungs and their relative weight were recorded.

### 2.7. Efficacy Study in Mouse Model of Malaria

#### 2.7.1. Parasite Inoculation and Amplification

*Plasmodium berghei* strain B (***Pb***B) (MRA-406, MR4, ATCC® Manassas Virginia) was obtained from BEI Resources (https://www.beiresources.org) and used for the experiments. A cryopreserved parasite was thawed and injected intraperitoneally (i.p.) into naïve recipient mice. Three days after infection, the parasitemia was monitored daily in tail vein blood through Giemsa-stained thin smear observation under oil immersion at 100x magnification. Passage of infection in new mice consisted of an i.p. injection of 200 *μ*L of 10^6^ infected erythrocytes/mL sterile PBS [20].

#### 2.7.2. Evaluation of the Curative Effect of the Aqueous Extract from *T. mantaly* Stem Bark (*Tm*sb^w^)

The antimalarial curative effect of *Tm*sb^w^ was assessed in malaria-infected mice as described by Fidock et al. [21]. Twenty-five female mice were inoculated with *Pb*B-infected erythrocytes, and 72 hours later, parasitemia was checked to confirm infection. Infected mice were then separated into five batches of five mice each. The three test batches received *Tm*sb^w^ at single daily oral dosages of 100, 200, and 400 mg/kg for 5 consecutive days, starting day 3 (72 hours) postinfection. The negative control and positive control groups received, respectively, a daily dose of 10 mL/kg of distilled water and 10 mg/kg of chloroquine, respectively. A sixth group of five uninfected female mice considered as the normal group was monitored without any treatment in the same experimental condition. Parasitemia was followed daily from tail blood of mice and Giemsa staining. The percent parasitemia obtained 24 h after the last drug administration was used to determine the dose that reduce parasitemia to 50% or effective dose 50 (ED_50_) using GraphPad Prism 5.0 software. The percent parasitemia was calculated daily using the following formula [21]:

(1) $$\begin{array}{c} \%\text{Parasitemia}=\frac{\text{Number of parasitized RBC}}{\text{Total number of RBC}}\times 100. \end{array}$$

The percent of parasitemia chemosuppression of *Tm*sb^w^ was calculated at the end of the studies and compared with respect to the controls. Parasitemia chemosuppression was calculated using the following formula [21]:

(2) $$\begin{array}{c} \%\text{Chemosuppression}=\frac{\text{Mean of parasitemia of untreated group}-\text{Mean of parasitemia of treated group}}{\text{Mean of parasitemia of untreated group}}\times 100. \end{array}$$

#### 2.7.3. Hematological, Biochemical, and Histopathology Analyses

At the end of the assays, animals were anesthetized using urethane and blood samples were collected by cardiac puncture in heparin and dried tubes for hematological and biochemical analysis, respectively. Serum was obtained by blood centrifugation at 3000 rpm for 15 min and stored at -20°C for the biochemical parameters' analysis, with focus on transaminase enzymes ALT and AST using Kits Hospitex. Blood in heparinized tubes was used for hematological parameter analysis using autoanalyzer Cell-Dyn Model 331 430. Histopathological analysis of the liver was performed according to the protocol described by Pieme et al. [22]. In brief, mouse liver pieces (3–5 *μ*m thick) were fixed in 10% Formalin (Sigma-Aldrich, Germany) for 24 h and washed in running water for 24 h. Samples were dehydrated in a cup containing increasing concentration of ethanol at different times and then cleared in two cups containing xylene during 1 h and 1.5 h, respectively, to remove absolute alcohol. Embedding was done by passing the cleared samples through three cups containing molten paraffin at 50°C and in a cubical block of paraffin. It was followed by microtome cutting. The slides were stained using hematoxylin-eosin for microscopic examination.

### 2.8. Statistical Analysis

The ED_50_ and IC_50_ were determined using GraphPad Prism 5.0 software while hematological and biochemical data were analyzed using STATGRAPHICS Version 5.0. Statistical significance testing was done using Least Significant Difference followed by Fisher's test (ANOVA). *P* values of less than 0.05 were considered statistically significant. Data were then expressed as mean ± standard deviation (SD).

## 3. Results and Discussion

### 3.1. Extraction Yield and Phytochemical Composition

Crude plant extracts were prepared from the leaves and stem bark with extraction yields calculated with respect to the dried vegetal material. The extraction yields ranged from 1.56% to 29.5% with respect to the plant part and extraction solvent (Table 1).

Water, methanol, hydroethanol, and ethanol presented the best extraction yield *T. mantaly* as per plant part. In fact, distilled water, methanol, hydroethanol, and ethanol are polar solvents and will mainly extract polar phytochemicals.

The phytochemical screening revealed the presence of alkaloids, phenolics, glucosides, triterpenes, saponins, and flavonoids in all the crude plant extracts.

### 3.2. *In Vitro* Antiplasmodial Activity of *T. mantaly* Plant Extracts

Globally, 7 out of the 12 extracts prepared showed activity against *Pf*W2 with their IC_50_ values ranging from 0.809 *μ*g/mL to 5.886 *μ*g/mL. Five extracts showed very good antiplasmodial activity (IC_50_ < 5 *μ*g/mL) and two exerted moderate antiplasmodial activity (5 *μ*g/mL ≤ IC_50_ < 10 *μ*g/mL) [7]. Four extracts out of the eight more potent (IC_50_ < 5 *μ*g/mL) antiplasmodial extracts were prepared with distilled water and methanol (three extracts each), suggesting that their antiplasmodial constituent of *Terminalia mantaly* are polar. Indeed, the aqueous extracts were more active than hydroethanolic and ethanolic extracts; this might justify the traditional choice of water to prepare phytodrugs from *Terminalia mantaly* against malaria [10, 23].

From the six extracts prepared from the leaves of *T. mantaly*, four (66.67% of extracts) exhibited antiplasmodial activity with their IC_50_ varying from 2.203 *μ*g/mL to 5.886 *μ*g/mL. Three extracts, namely, *Tm*l^w^ (IC_50_ = 2.203 *μ*g/mL), *Tm*l^m^ (IC_50_ = 2.367 *μ*g/mL), and *Tm*l^d^ (IC_50_ = 4.036 *μ*g/mL), were very active (IC_50_ < 5 *μ*g/mL) against *P. falciparum* W2 whereas the fourth one showed moderate antiplasmodial activity with an IC_50_ value of 5.886 *μ*g/(*Tm*l^h-e^). Three extracts out of the six prepared from the stem bark of *T. mantaly* showed activity against *Pf*W2 with the values of IC_50_ ranging from 0.809 *μ*g/mL to 5.215 *μ*g/ml. *Tm*sb^w^, aqueous extract of the stem bark, presented the highest antiplasmodial activity (IC_50_ < 1 *μ*g/mL) with an IC_50_ value of 0.809 *μ*g/mL, followed by *Tm*sb^m^ (IC_50_ = 2.062 *μ*g/mL) and *Tm*sb^d^ (IC_50_ = 5.215 *μ*g/mL). Moreover, extracts from the stem bark of *T. mantaly* were more active than those from *T. brownii* investigated by Machumi et al. [24] who reported antiplasmodial activity of ethyl acetate extract (IC_50_ < 5.3 *μ*g/mL) and aqueous extract (IC_50_ = 27.4 *μ*g/mL) against *Pf*W2.

Globally, the aqueous extract from the stem bark of *T. mantaly* presented the highest antiplasmodial activities *in vitro* on both resistant and sensitive strains of *P. falciparum* with IC_50_*Pf*W2 = 0.809 *μ*g/mL (Table 1) in line with that obtained by Mbouna et al. with IC_50_*Pf*INDO = 0.26 *μ*g/mL and IC_50_*Pf*3D7 = 1.03 *μ*g/mL [13]. The stem bark of *T. mantaly* was further submitted to oral acute toxicity and efficacy evaluation in malaria rodent model.

### 3.3. Acute Toxicity Profile of *Tm*sb^w^

#### 3.3.1. Effect of *Tm*sb^w^ on Mouse General Behavior

No death or adverse effects were observed in animals after oral administration of a single dose of *Tm*sb^w^ (2000 mg/kg) over 14 days.

#### 3.3.2. Effect of *Tm*sb^w^ on Mouse Body Weight

**Figure 1:**
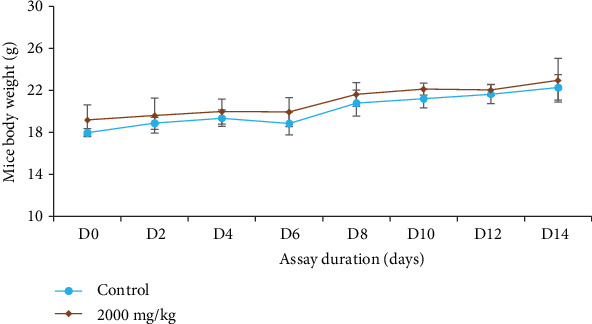
Evolution of mouse body weight with respect to experimentation duration.

*Tm*sb^w^ at 2000 mg/kg prevented a reduction in body weight of test mice compared to control ones (Figure 1). In the same line, studies conducted by Kamo et al. [9] showed no sign of toxicity after oral administration of the hydroalcoholic extract from *T. mantaly* stem bark at the doses of 2000 and 5000 mg/kg. Taking together this study with the one conducted by Kamo et al. [9] highlights the safety of *T. mantaly* plants extracts against nontarget cells in the human body no matter the solvent used.

### 3.4. Curative Antimalarial Effect of *Tm*sb^w^

#### 3.4.1. Curative Effect of *Tm*sb^w^

**Figure 2:**
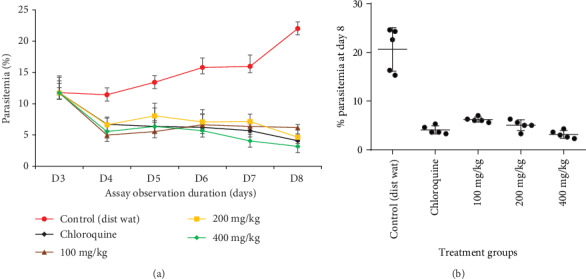
Curative effect of *Tm*sb^w^ as compared to chloroquine (CQ) and the negative control (NC). (a) Effects of *Tm*sb^w^ on the parasitemia of infected mice during the whole treatment time. (b) Effect of *Tm*sb^w^ on the parasitemia of infected mice on day 8.

Figure 2 shows the chemosuppressive activity of *Tm*sb^w^ on parasitemia of infected mice.

The results indicated that intraperitoneal inoculation of mice with 10^6^ of *Pb*B-parasitized red blood cells (RBC) led to malaria infection with parasitemia up to 22.03% at the end of experimentation in the negative control group (Table 2). *Tm*sb^w^ exhibited significant (*P* < 0.05) dose-dependent reduction of parasitemia compared to the negative control after oral administration for five days to infected mice (Table 2) highlighting the inhibitory effect of *Tmsb^w^* on malaria parasite life cycle in this model.

*Tm*sb^w^ at the 8^th^ day postinfection reduced the parasitemia by, respectively, 71.93%, 80.97%, and 85.64% at 100, 200, and 400 mg/kg doses. *Tm*sb^w^ effective dose that reduce 50% of parasitemia (ED_50_) was 69.50 mg/kg. *Tm*sb^w^ exerted dose-dependent antimalarial curative effect, closer to that of the chloroquine especially at 400 mg/kg/day. Extracts that displayed a parasitemia suppression percent greater than or equal to 50% at 500, 250, or 100 mg/kg of body weight daily are classified as having moderate, good, or very good antimalarial activity, respectively [25]. At 100 mg/kg/day dosage, *Tm*sb^w^ displayed very good antimalarial potency by significantly reducing the parasitemia above 50% (Table 2, 71.93%) and could be considered as having very good antimalarial activity .

Overall, the high *in vitro* and *in vivo* antimalarial potency of *T. mantaly* and particularly the stem bark extract *Tm*sb^w^ could be due to its rich and diversified phytochemicals. Indeed, recent investigations on phytochemical analysis of *T. mantaly* revealed the presence of several phytochemical families, including mainly alkaloids, phenols, flavonoids, tannins, saponins, and steroids. Moreover, many compounds belonging to these classes of phytochemicals have been studied and reported as highly potent against several sensitive and resistant strains of *P. falciparum*, *P. berghei*, *P. chabaudi chabaudi*, and *P. vinckei petteri* [13, 20, 26–28].

#### 3.4.2. Effect of *Tm*sb^w^ on Mouse Relative Organ Weight

**Figure 3:**
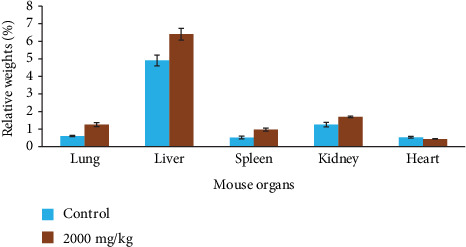
Evolution of mouse relative organ weight following treatment with *Tm*sb^w^. Bars represent means ± SD, *n* = 5.

*Tm*sb^w^ at a single dose increased significantly (*P* < 0.05) the relative weight of the lung, liver, kidney, and spleen in the test group compared to the control group (Figure 3), highlighting potential inflammation of mouse organs following *Tm*sb^w^ administration.

Overall, *Tm*sb^w^ showed nonvaluable toxic effects at up to 2000 mg/kg/day when orally administrated to mice and was considered safe with an LD_50_ greater than 2000 mg/kg [19, 24].

#### 3.4.3. Effect of *Tm*sb^w^ on Infected Mouse Body Weight

**Figure 4:**
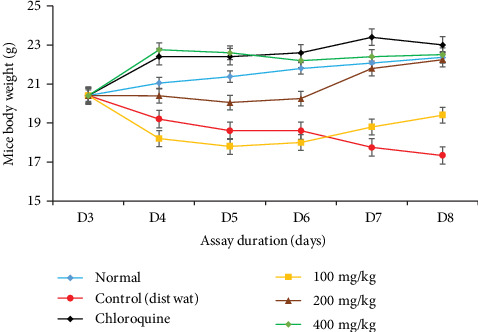
Evolution of body weight of infected mice during the treatment with *Tm*sb^w^.

The effect of *Tmsb^w^* on mouse body weight following infection was monitored, and the results showed that *P. berghei* infection induced a significant decrease of body weight in infected untreated mice (control) compared to normal and infected treated animals (Figure 4).

Daily administration of *Tm*sb^w^ for five consecutive days significantly protected infected mice from body weight loss compared to negative control animals (Figure 4), where significant decrease of body weight was observed from day 7 postinfection. No difference was observed in the positive control and normal groups compared to the test groups. This result is in line with the findings of Haidara et al. [26] who reported no weight loss after treatment of *P. berghei-*infected mice with closely related species, *Terminalia macroptera* extracts.

#### 3.4.4. Effect of *Tm*sb^w^ on Hematological Parameters

Results of the hematological analysis are summarized in Table 3.

Malaria infection induced significant decrease of total RBC counts, hemoglobin (HGB), and hematocrit (HCT) rates by 41.69%, 58.93%, and 60.23%, respectively, in untreated infected mice compared to normal (uninfected mice) group. Daily administration of *Tm*sb^w^ led to significant dose-dependent increase in HGB by 22.18%, 66.81%, and 84.82% at 100, 200, and 400 mg/kg doses, respectively, compared to the negative control group. The total white blood cell (WBC) count significantly increased in the negative control compared to uninfected mice. *Plasmodium* species are intraerythrocytic parasites of vertebra host, where they intake in the cytoplasm or on the surface of the target cell, the substance essential for their growth [25]. This life cycle of parasite contributes at the end of each cycle at the rupture of RBC, reduction of hemoglobin and hematocrit. *Tm*sb^w^ treatment at various doses of the assay limited the adverse effect of parasite indicating its potential protective effects on hematological parameters of infected mice.

#### 3.4.5. Effect of *Tm*sb^w^ on Some Biochemical Parameters

Table 4 summarizes the effects of *Tm*sb^w^ on transaminases of *Pb*B-infected mice.

Malaria infection caused by *P. berghei* significantly increased ALT and AST activities in untreated mice compared to uninfected mice. Daily treatment with *Tm*sb^w^ significantly prevented ALT and AST activity increase in infected mice by 27.03%, 18.45%, and 64.80% (ALT) and by 88.29% and 92.54% (AST) at increasing doses of extract, respectively (Table 4).

#### 3.4.6. Effect of *Tm*sb^w^ on Liver Histology

**Figure 5:**
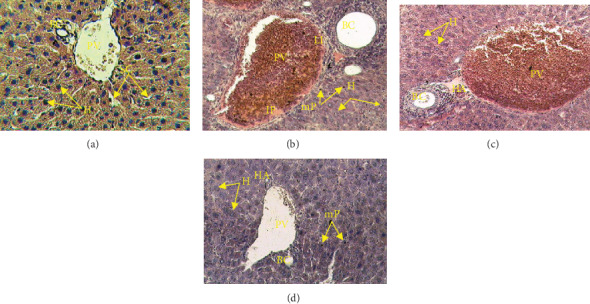
Photomicrograph of the effects of *Tm*sb^w^ on the histology of mouse liver infected by *Pb*B: (a) uninfected mice, (b) untreated mice, (c) chloroquine-treated mice, and (d) *Tm*sb^w^-treated mice; HE ×400. HA: hepatic artery; BC: biliary canal; PV: portal vein; H: hepatocyte; SC: sinusoid capillary; H: dilate hepatocyte; LI: leucocyte infiltration; mP: malarial pigment; FI: inflammatory focal point.

The liver of infected mice showed vascular congestion and leucocyte infiltration (Figure 5). Malarial pigment was observed in liver tissue (Figure 5(b)), indicating infection of mouse hepatocytes by *Pb*B. This vascular congestion was significantly reduced in *Tm*sb^w^-treated mice compared to untreated control. The architecture of the liver of *Tm*sb^w^-treated mice was like that of uninfected ones (Figure 5(d)). Overall, no significant damage was observed in liver tissues of *Tm*sb^w^-treated mice compared to healthy mice.

The liver is the main functional organ where ALT and AST activities take place. *P. berghei*-infected and untreated mice showed a hepatomegaly and an increase of the liver enzymes AST and ALT indicating an alteration of some hepatic metabolic functions [29, 30]. Daily oral administration of *Tm*sb^w^ induced the decrease of the enzyme activities highlighting its potential protective effect on liver damages caused by *P. berghei*.

## 4. Conclusion

The findings achieved in this study provide evidence that *Tm*sb^w^, the aqueous extract from the stem bark of *T. mantaly*, is relatively safe and highly potent on *P. berghei-*infected mice and thereby validate the use of *T. mantaly* in folk medicine to treat malaria and related symptoms. However, *Tm*sb^w^ should be further investigated to isolate and characterize its bioactive principle to feed antimalarial drug discovery pipeline.

## Figures and Tables

**Table 1:** Extraction yields and antiplasmodial activity of *T. mantaly* crude extracts.

Species	Plant parts	Solvents	Codes	Yields (%)	IC_50_ ± SD (*μ*g/mL)
*T. mantaly*	Leaves	Water	*Tm*l^w^	23.11	2.203 ± 0.898
		Methanol	*Tm*l^m^	29.50	2.367 ± 0.483
		Hydroethanol	*Tm*l^h-e^	27.71	5.886 ± 0.070
		Ethanol	*Tm*l^e^	11.51	>10.00
		DCM	*Tm*l^d^	21.00	4.036 ± 0.150
		Hexane	*Tm*l^h^	01.56	>10.00
	Stem bark	Water	*Tm*sb^w^	19.57	0.809 ± 0.258
		Methanol	*Tm*sb^m^	22.47	2.062 ± 0.292
		Hydroethanol	*Tm*sb^h-e^	29.08	>10.00
		Ethanol	*Tm*sb^e^	25.45	>10.00
		DCM	*Tm*sb^d^	14.12	5.215 ± 0.040
		Hexane	*Tm*sb^h^	2.00	>10.00

Artemisinin (*μ*M)					0.005 ± 0.001

Data are presented as means of triplicate experiments. IC_50_: 50% inhibitory concentration; SD: standard deviation from triplicate experiments; *Tm*: *Terminalia mantaly*; l: leaf; sb: stem bark; r: roots. ^w^Water, ^m^methanol, ^h-e^hydroethanol 70%, ^e^ethanol, ^d^dichloromethane (DCM), and ^h^hexane.

**Table 2:** Curative effect of *Tm*sb^w^ on *P. berghei*-infected mice.

Treatment ↓	Dose	Parasitemia (%)	Chemosuppression (%)
Distilled water (negative control)	10 mL/kg	22.02 ± 2.58	—
*Tm*sb^w^ (*T. mantaly* stem bark aqueous extract)	100 mg/kg	6.18 ± 0.52	71.93 ± 0.79
	200 mg/kg	4.19 ± 1.19	80.97 ± 0.53
	400 mg/kg	3.16 ± 0.80	85.64 ± 0.68
Chloroquine (positive control)	10 mg/kg	4.08 ± 0.84	81.47 ± 0.67
ED_50_*Tm*sb^w^ = 69.50 mg/kg			

ED_50_: 50% effective dose; *Tm*: *Terminalia mantaly*; sb: stem bark. ^w^Water.

**Table 3:** Effects of *Tm*sb^w^ on some hematological parameters in *Pb*B-infected mice.

Parameters	Controls			*Tm*sb^w^ (daily oral dosage)		
	Normal (uninfected)	Negative (untreated)	Positive (chloroquine)	100 mg/kg	200 mg/kg	400 mg/kg
RBC (10^3^/mm^3^)	6.38 ± 1.97^b^	3.72 ± 0.97^a^	4.19 ± 0.83^a^	4.04 ± 0.61^a^	4.52 ± 1.33^a^	4.97 ± 1.37^a^
HGB (g/dL)	14.04 ± 1.62^a^	8.83 ± 1.50^b^	10.51 ± 2.97^a^	11.36 ± 0.81^a^	14.73 ± 5.64^a^	16.32 ± 6.09^a^
HCT (%)	72.92 ± 9.08^b^	29.00 ± 6.98^a^	33.36 ± 6.27^a^	32.37 ± 4.31^a^	36.8 ± 4.61^a^	37.56 ± 7.14^a^
MCV (fL)	97.33 ± 7.02^a^	87.17 ± 10.22^a^	86.28 ± 9.64^a^	82.45 ± 5.18^a^	77.82 ± 10.91^a^	82.04 ± 13.29^a^
MCH (pg)	23.52 ± 5.88^a^	67.57 ± 18.85^b^	46.40 ± 2.25^a^	42.46 ± 13.48^a^	68.05 ± 10.91^b^	30.53 ± 0.57^a^
MCHC (g/dL)	19.22 ± 1.24^a^	38.18 ± 3.90^b^	34.40 ± 7.19^a^	31.95 ± 13.48^a^	28.22 ± 2.00^a^	31.53 ± 3.12^a^
WBC (10^3^/*μ*L)	11.50 ± 3.30^a^	14.30 ± 2.07^b^	5.84 ± 2.10^a^	21.84 ± 2.07^a^	24.75 ± 1.46^a^	21.70 ± 3.83^a^
LYM (10^3^/mm^3^)	10.16 ± 2.83^b^	2.05 ± 0.31^a^	3.26 ± 0.71^a^	4.05 ± 1.34^a^	2.49 ± 0.92^a^	5.17 ± 1.83^a^
MON (%)	0.75 ± 0.17^b^	1.56 ± 0.48^a^	1.10 ± 0.32^a^	5.16 ± 0.22^a^	1.00 ± 0.29^a^	2.68 ± 0.75^a^
GRA (10^3^/mm^3^)	0.93 ± 0.23^b^	4.63 ± 0.71^a^	6.38 ± 1.91^a^	4.84 ± 1.92^a^	5.17 ± 0.73^a^	4.36 ± 0.73^a^
PLT (10^3^/mm^3^)	445 ± 109^a^	565 ± 123^a^	497 ± 91^a^	358 ± 127^a^	458 ± 114^a^	509 ± 160^a^

MCHC: mean corpuscular hemoglobin concentration; MCH: mean corpuscular hemoglobin; HCT: hematocrit; HGB: hemoglobin concentration; LYM: lymphocyte; MON: monocyte; GRA: granulocyte; MCV: mean corpuscular volume; RBC: red blood cell; PLT: platelet; WBC: white blood cell; *Tm*: *Terminalia mantaly*; sb: stem bark. ^w^Water. ^a, b^Statistical difference at *P* < 0.05 (*n* = 5); the same letters on the same line indicate no statistical differences. Obtained data were compared as follows: negative control with normal group and infected treated groups with negative control.

**Table 4:** Biochemical parameters of *Pb*B-infected mice treated with *Tm*sb^w^.

Parameters	Controls			*Tm*sb^w^ (daily oral dosage)		
	Normal (uninfected)	Negative (untreated)	Positive (chloroquine)	100 mg/kg	200 mg/kg	400 mg/kg
AST	212.0 ± 11.6^a^	233.0 ± 34.5^b^	55.0 ± 9.07^a^	170.0 ± 22.9^a^	190.0 ± 23.9^a^	82.0 ± 13.6^a^
ALT	89.0 ± 5.7^a^	657.0 ± 73.5^b^	27.0 ± 4.07^a^	590.0 ± 38.3^b^	61.0 ± 8.6^a^	49.0 ± 9.3^a^

AST: aspartate transaminase; ALT: alanine transaminase; *Tm*: *Terminalia mantaly*; sb: stem bark. ^w^Water. ^a, b^Statistical difference at *P* < 0.05; the same letters on the same line indicate no statistical differences.

## Data Availability

No data were used to support this study.
